# Neural mechanisms of emotional health in traumatic brain injury patients undergoing rTMS treatment

**DOI:** 10.1038/s41380-023-02159-z

**Published:** 2023-07-06

**Authors:** Tajwar Sultana, Muhammad Abul Hasan, Xiaojian Kang, Victoria Liou-Johnson, Maheen Mausoof Adamson, Adeel Razi

**Affiliations:** 1 Department of Computer and Information Systems Engineering, NED University of Engineering & Technology, Karachi, 75270 Pakistan; 2 Department of Biomedical Engineering, NED University of Engineering & Technology, Karachi, 75270 Pakistan; 3 Neurocomputation Laboratory, National Centre of Artificial Intelligence, Peshawar, Pakistan; 4 WRIISC-WOMEN, VA Palo Alto Healthcare System, Palo Alto, CA 94304 USA; 5 Rehabilitation Service, Veterans Affairs Palo Alto Healthcare System (VAPAHCS), 3801 Miranda Avenue, Palo Alto, CA 94304 USA; 6 Clinical Excellence Research Center, Stanford University School of Medicine, Stanford, CA 94304 USA; 7 Department of Neurosurgery, Stanford University School of Medicine, Stanford, CA 94304 USA; 8 Turner Institute for Brain and Mental Health, School of Psychological Sciences, Monash University, Clayton, VIC 3800 Australia; 9 Wellcome Centre for Human Neuroimaging, University College London, WC1N 3AR London, United Kingdom; 10 CIFAR Azrieli Global Scholars Program, CIFAR, Toronto, ON Canada

**Keywords:** Neuroscience, Diseases

## Abstract

Emotional dysregulation such as that seen in depression, are a long-term consequence of mild traumatic brain injury (TBI), that can be improved by using neuromodulation treatments such as repetitive transcranial magnetic stimulation (rTMS). Previous studies provide insights into the changes in functional connectivity related to general emotional health after the application of rTMS procedures in patients with TBI. However, these studies provide little understanding of the underlying neuronal mechanisms that drive the improvement of the emotional health in these patients. The current study focuses on inferring the effective (causal) connectivity changes and their association with emotional health, after rTMS treatment of cognitive problems in TBI patients (*N* = 32). Specifically, we used resting state functional magnetic resonance imaging (fMRI) together with spectral dynamic causal model (spDCM) to investigate changes in brain effective connectivity, before and after the application of high frequency (10 Hz) rTMS over left dorsolateral prefrontal cortex. We investigated the effective connectivity of the cortico-limbic network comprised of 11 regions of interest (ROIs) which are part of the default mode, salience, and executive control networks, known to be implicated in emotional processing. The results indicate that overall, among extrinsic connections, the strength of excitatory connections decreased while that of inhibitory connections increased after the neuromodulation. The cardinal region in the analysis was dorsal anterior cingulate cortex (dACC) which is considered to be the most influenced during emotional health disorders. Our findings implicate the altered connectivity of dACC with left anterior insula and medial prefrontal cortex, after the application of rTMS, as a potential neural mechanism underlying improvement of emotional health. Our investigation highlights the importance of these brain regions as treatment targets in emotional processing in TBI.

## Introduction

Traumatic brain injury (TBI) is frequently characterized as a silent epidemic because of its high rate of incidence and dire consequences [1]. According to the memorandum issued by the US Department of Defense, in 2015, the TBI severity stratification into mild, moderate, and severe is based on duration of unconsciousness, duration of alteration of consciousness and post traumatic amnesia [2]. TBI is an event where after the initial injury, a pathophysiological process begins that generates structural and functional alterations leading to cognitive, social, and behavioral deficits [3].

Usually, mild TBI (mTBI) patients sustain long-term neuropsychiatric disorders [4] in addition to impairments in cognitive domains such as attention, memory and executive control [5, 6]. It is crucial to manage these long-term implications to improve the patients’ quality of life [7]. The intervention mechanisms include, but are not limited to, pharmacotherapy, psychotherapy, and non-invasive brain stimulation techniques. Various modes of intervention are prescribed at different stages of TBI recovery. At the acute and subacute stage, controlling the neurochemical disturbances are desirable to promote survival probability and resist the functional disability. At the chronic stage non-invasive rehabilitation techniques are used to address the change in neuroplasticity following TBI and to promote reorganization of neural network for recovery [8]. Repetitive transcranial magnetic stimulation (rTMS) is a well-recognized therapeutic alternative for brain function modulation. It is a non-invasive method to stimulate specific brain regions by applying an intermittent magnetic field using an electromagnetic coil. It is an FDA-approved method for the treatment of depression and Obsessive Compulsive Disorder (OCD) but its use in TBI is still under investigation [9–12].

The neuroimaging modality most widely employed to assess and monitor the functional modulation in TBI patients is functional magnetic resonance imaging (fMRI). Numerous research studies have been conducted to investigate the impact of modulation on brain functional connectivity post-TBI using fMRI [13–16]. Resting-state fMRI analysis has also been extensively performed to understand the baseline brain connectivity of healthy and TBI populations. The major brain networks and/or regions studied during previous studies in TBI include the core default mode network (DMN) [13, 17–23], medial temporal lobe (MTL) [21, 24] anterior cingulate cortex [25, 26], amygdala [21, 27], insula [26], thalamus [21, 26] and other subcortical regions. The most studied brain network in TBI is DMN. DMN is the brain network that shows increased activation in the awake mode without any externally oriented task [28]. It comprises different subsystems including mPFC, PCC, and medial temporal lobe (MTL). Mental health disorders including anxiety, stress, and depression are frequently observed in TBI patients which is a major hindrance in their recovery and consequently leads to cognitive disorders and social abnormalities [29]. Increased DMN connectivity [22], increased ACC connectivity [25], and increased amygdala connectivity [27] in resting-state may be regarded as biomarkers in chronic TBI with comorbid mental health disorders. Elevated aggression level was associated with increased resting-state connectivity between the right hippocampus and midcingulate cortex [30]; other regions affected by depression in TBI include insula, thalamus, and ACC [26].

The mental health sequelae of TBI including emotional dysfunction have a strong impact on the quality of life and daily life functioning of the patient [31]. There is an urgent need of sustained research to identify the effective rehabilitation techniques [32]. There is a growing interest in TMS as a treatment for post-concussive symptoms specifically depression [33–38], however, previous research in rTMS concerning prognosis, biomarker identification and investigation of underlying neural mechanisms (using effective connectivity) was mostly focused on psychiatric disorders. There are only a few studies assessing brain functional connectivity changes in treating psychological deficits in TBI using rTMS [36, 39]. The results of these studies have given insight into the changes in functional connectivity between brain regions; however, they do not provide underlying neuronal mechanisms that generate them. Despite the importance of effective connectivity in formulating the pathways for post-concussive symptoms therapy, virtually no research exists. The current work is the first that uses effective connectivity to investigate the utility of using rTMS with TBI. This study focuses on inferring the changes in effective connectivity observed in TBI patients after treatment with rTMS and the association of effective connectivity with the emotional health assessment. Dynamic causal modeling [40, 41] (DCM) is the preferred approach for the analysis of effective connectivity using multivariate neural time series from various regions of interest. We used its variant called spectral DCM [42, 43] (spDCM) which is widely adopted to model the directed communication among brain regions in the resting state. Previous work has shown the reliability of the resting state effective connectivity estimation using spDCM [44]. We selected the distributed brain regions influencing emotional well-being after TBI, using evidence from previous literature [45–47]. These brain regions include the anterior and posterior hubs of DMN; mPFC, and PCC, the hippocampus which is the hub of the medial temporal lobe, and the salience network regions: dACC, AI and AMG. We also selected the target-site of rTMS and its counterpart across hemispheres that are the bilateral DLPFC which are part of the Executive Network (EN). Our aim is to investigate the resting state causal connectivity among distributed brain regions of chronic TBI subjects related to emotional network before and after the therapeutic intervention of rTMS. We hypothesize that treatment with rTMS would yield improved emotional health in TBI subjects, and based on the previous functional connectivity literature, the effective connectivity between the selected tri-network would play major a role in the emotional wellness.

## Materials and methods

### Dataset

The anonymized dataset consisted of 32 veterans with TBI who were recruited from Veterans Affairs Palo Alto Health Care System (VAPAHCS) and surrounding community via advertisements. The experimental protocol of the double-blind randomized clinical trial was approved by the Institutional Review Boards (IRB) of Veterans Affairs Palo Alto Health Care System (VAPAHCS) and Stanford University. The age range of participants was from 20 to 69 years with 27 males and 5 females. The severity level of TBI was either mild or moderate for each participant. The participants were split up into active (*N* = 17) and sham (*N* = 15) groups randomized on mild and moderate TBI. The data was divided into 3 sets: baseline (pre-rTMS), immediately after the end of the treatment period (post-rTMS), and at six months follow-up. The current analysis only utilized the dataset acquired at the first two instances. The rTMS treatment period of 20 sessions comprised of 2 weeks with 2–3 treatments per day. The rTMS was delivered to active group participants at left DLPFC with 80 trains of 5 sec each at 10 Hz frequency and the inter-train interval was 10 s. The sham group was provided with a similar setup as active participants except they were not given rTMS. For complete details about the data, please refer to Adamson et al. [10] and supplementary material.

The MRI and rs-fMRI data were acquired using GE 3 T MRI scanner from VAPAHCS. The acquisition parameters for structural scan were TR = 7.24 ms, TE = 2.78 ms, flip angle = 12°, voxel size 0.9 × 0.9 × 0.9 mm, 192 axial slices. The functional images were collected with parameters: TR = 2000 ms, TE = 30 ms, flip angle = 77°, FOV = 232 mm, voxel size 2.9 × 2.9 × 2.9 mm, 42 axial slices, 250 frames in 8 m 20 s.

### Emotional health assessment

A neuropsychological assessment battery was also executed on each patient which included the Veterans RAND 36 Item Health Survey (VR-36) at baseline, post-rTMS and follow-up to assess the physical and mental health. The mental health subscale (8th scale) of VR-36 also known as emotional well-being [48], consists of five items (Supplementary Table S1) that spans four major mental health categories including anxiety, depression, loss of behavioral or emotional control and psychological well-being [49]. The scoring process consists of two steps, coding and re-coding of responses [48]. All questions for the emotional health assessment have a response category (codes) from 1 to 6 (Supplementary Table S2). Each item’s score is re-coded on a range of 0 to 100 with 0 and 100 representing the lowest and highest scores respectively (Supplementary Table S3). The re-coded scores of all five items are averaged to generate the emotional health score.

### Preprocessing

**Fig. 1:**
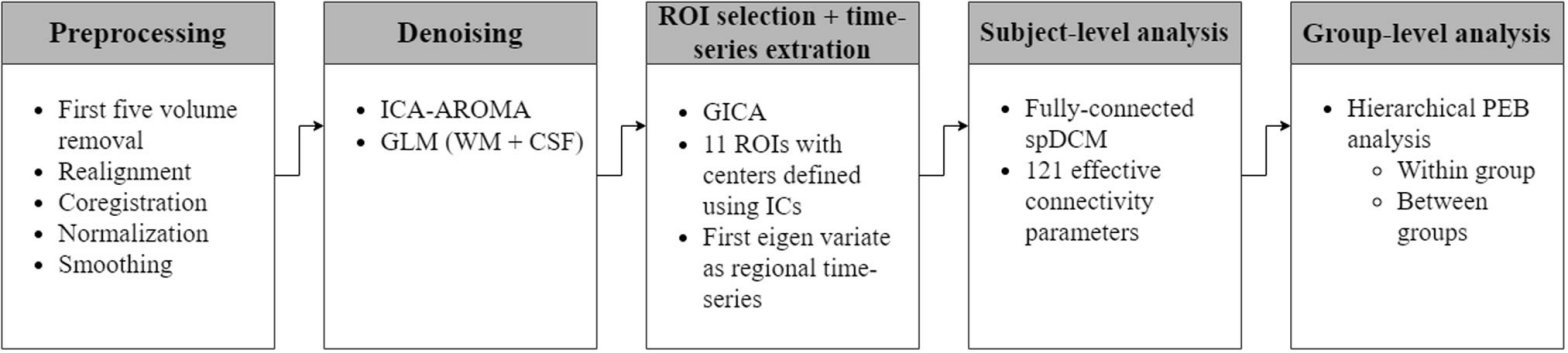
Methods overview. Flowchart depicting the pipeline of analysis methods.

The preprocessing and subsequent subject-level and group-level analysis were performed using Statistical Parametric Mapping software (SPM 12). The stepwise complete analysis is depicted using a flowchart in Fig. 1. The preprocessing steps included DICOM to NIfTI conversion, removal of first five volumes, realignment of the brain slices using rigid-body transformation with six parameters (3 translational and 3 rotational), coregistration of the structural and functional images, segmentation of MRI images according to their tissue types using tissue probability maps, normalization of structural and functional images to the standard MNI coordinate system using affine transformation, and smoothing of functional images using 6 mm full-width half-maximum Gaussian kernel. After spatial preprocessing, the denoising of the dataset was performed using ICA-based automatic removal of artifacts (ICA-AROMA) [50] and performing nuisance regression using general linear model (GLM) with white matter (WM) and cerebrospinal fluid (CSF) time series as regressors.

### ROI selection and time series extraction

The preprocessed data were then used to obtain a set of independent components to identify the desired resting-state networks (RSNs; DMN, SN, ECN) and to define the peak coordinates of 11 regions of interest namely posterior cingulate cortex (PCC), medial prefrontal cortex (mPFC), bilateral hippocampus (HP), bilateral amygdala (AMG), dorsal anterior cingulate cortex (dACC), bilateral anterior insula (AI) and bilateral dorsolateral prefrontal cortex (DLPFC). Spatial ICA was performed using group independent component analysis fMRI toolbox (GIFT) [51] and 75 independent components were estimated. A two-step principal component setup was executed to extract 100 subject-specific Principal Components (PCs) and 75 PCs from the aggregate data. Subject-specific spatial maps and time courses were estimated using the GICA back-reconstruction algorithm. The built-in tool ICASSO was used to run the Infomax algorithm 20 times to ensure the reliability of components. The components were scaled using the z-scores. The 75 ICs were then spatially correlated with (default) RSN templates which contain 90 functional ROIs across 14 large-scale RSNs [52] to identify the intrinsic brain networks. The peak MNI coordinates (using xjview [https://www.alivelearn.net/xjview]) in the resulting components were then used as the center of spheres for the desired regions (Table 1). The spherical regions were specified with radius of 8 mm. Additionally, binary masks were used for the regions which are smaller in area which were created using AAL [53] for amygdala and anterior insula and; RSN template masks for hippocampus [52]. Then the first principal component of the voxels time series was extracted from each spherical region to be used in DCM analysis. In the first study, the stimulation site for TMS, left DLPFC, was selected using neuronavigation [10] as shown in Supplementary Fig. S1; detailed information is available in the Supplementary material. For connectivity analysis in the current study, the DLPFC centroid was selected based on the same principle as for other ROIs - peak MNI coordinates in the independent components - to preserve consistency in methodology.

**Table 1:** MNI Coordinates of the centers of spheres of ROIs.

ROI	x	y	Z	Network
PCC	0	−43	23	DMN
mPFC	0	62	5	DMN
lHP	−21	−22	−16	DMN
rHP	24	−19	−16	DMN
lAMG	−18	−4	−16	SN
rAMG	18	−4	−16	SN
dACC	0	32	23	SN
lAI	−39	14	2	SN
rAI	39	17	2	SN
lDLPFC	−48	32	14	EN
rDLPFC	30	53	29	EN

### Effective connectivity analysis

The fMRI experiment was a 2 × 2 factorial design with factors at the level of treatment timings (pre-rTMS and post-rTMS) and intervention groups (active and sham). All 32 subjects were scanned before rTMS treatment (which we call here as pre-rTMS group) and out of them only 25 were scanned after completing rTMS treatment (which we call here as post-rTMS group). At the subject (first) level analysis, within subject connectivity was estimated using spectral dynamic causal modeling (spDCM) [42, 43, 54] and these connectivity parameters were then taken to group (second) level analysis to estimate the between group (pre-rTMS vs post-rTMS) connectivity parameters.

#### Subject-level analysis using spectral dynamic causal modeling

The effective connectivity of each subject in groups, pre-rTMS (active and sham) and post-rTMS (active and sham) was estimated using spDCM. It is a variant of DCM for the resting-state data based on the second-order statistics (cross spectra) of observed BOLD time series. It performs the modeling using cross-spectra; the frequency domain equivalent of the cross-correlation among time series [41, 42, 55]. (Please see supplementary material for technical description of spectral DCM). This analysis involved the specification of a fully connected model with 11 nodes (ROIs). To ensure the data fit, the cross spectral density plots were inspected visually resulting in 4 subjects being discarded from each pre- and post-rTMS groups due to bad models fits or large amount of residual noise. The cross spectral density of the bold signal follows the power-law distribution with a peak at very low frequency. However, after model inversion, for few subjects, the estimated peaks of regional cross spectral power had a peak at much higher frequencies (perhaps representing an aliased BOLD signal) which may occur if the signal was not properly cleaned for various artifacts. Based on this, only two subjects from active group were excluded at only post TMS while they were included in the analysis of the pre-TMS session. All other discarded data belonged to the same subjects in pre- and post-TMS groups.

#### Group-level analysis using hierarchical PEB

At the group level, a two-level hierarchical parametric empirical Bayes (PEB) was used [56, 57]: within group analysis (within pre-rTMS and within post-rTMS) at the first PEB level and between group analysis (post-rTMS vs pre-rTMS) at the second PEB level.

PEB is a statistical method that combines information (probability densities of subject-level parameters) across multiple subjects to estimate the group-level parameters. This involves specifying a Bayesian GLM incorporating the within-subject connectivity parameters’ probability densities as responses, and the between-subject or group-level parameters as covariates. The covariates usually represent the commonalities and differences across the subjects. The Bayesian model inversion of the ensuing PEB model provides the posterior estimates of the group-level connectivity parameters.

This technique is better than the classical approaches of statistical analysis (such as ANOVA) because the full posterior probability density (i.e. both the mean and the variance) of each subject-level parameters is carried to the group level. Since PEB uses the quantified uncertainty in addition to the mean value, which is in contrast to the classical inference, hence resulting in a statistically more powerful and robust inference. For the more detailed technical description, please refer to the supplementary material.

### Relationship of post-rTMS and pre-rTMS connectivity with emotional health

We used PEB for the association analysis between behavioral scores and connectivity by defining the connectivity as the response variable and scores as the regressor of interest. A PEB was defined to find the association between connectivity and emotional health scores in the post-rTMS group. The association analysis was conducted on both active and sham groups separately. In these PEB analyses only those DCM connections were considered which showed connectivity differences between pre- and post-rTMS in active group.

We also performed the association analysis of the effective connectivity of the entire cohort of pre-rTMS group (i.e., combined active and sham groups) with the baseline behavioral scores of the entire cohort.

## Results

At the group level connectivity and association analyses, the reported results are only those connections whose posterior probability (*pp*) >0.95. In the mean connectivity matrices, the positive and negative signs show excitatory and inhibitory connections respectively while in the difference connectivity matrices, the positive and negative sign represent the increase and decrease in the connectivity. Below, we only report results for the active group; sham group results are reported in the supplementary material.

### Behavioral analysis

The difference between the emotional health scores of active group pre- and post-rTMS were statistically significant (*p* = 0.0114; pre-rTMS - mean = 57.82, st. dev = 9.36, post-rTMS - mean = 73.09, st. dev = 16.60) showing emotional health improvement post-rTMS, while that of sham group were not statistically significant (*p* = 0.5126; pre-rTMS - mean = 70.40, st. dev = 16.61, post-rTMS - mean = 70.85, st. dev = 15.95). Please see supplementary information for analysis on executive function assessment.

### Connectivity difference between post rTMS and pre rTMS in active group

**Fig. 2:**
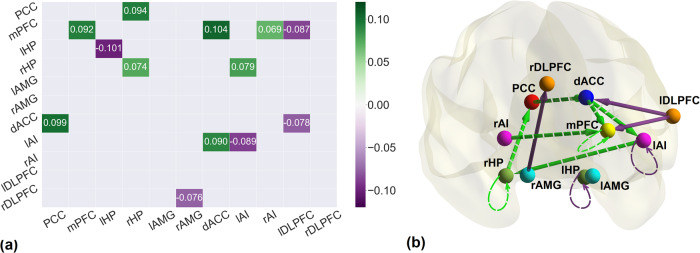
Effective connectivity changes after treatment with rTMS. **a** The connectivity matrix representing the difference between pre-rTMS and post-rTMS connectivity in active group. The green gradient illustrates increase while purple gradient depicts decrease in connectivity. **b** The brain diagram of the connectivity difference between pre-rTMS and post-rTMS in active group. Nodes represent the brain regions, edges represent the effective connectivity, green and purple arrows show increase and decrease in connectivity while solid and dashed lines represent excitatory and inhibitory connections respectively. All the connection here are in units of Hertz (Hz), except the self-connections which are log-scaled. All the connections reported here survived the threshold of posterior probability >0.95 amounting to a strong evidence.

Effective connectivity differences, in the active group, before and after the application of rTMS are shown in Fig. 2. The brain networks were visualized with the BrainNet Viewer [58]. The change in effective connectivity (increase or decrease) and the valence of connections (excitation or inhibition; reported using the mean connectivity shown in Supplementary Fig. S2) are reported in Table 2.

**Table 2:** Effective connectivity in active group (post rTMS – pre rTMS).

From	To	Excitatory (+) Inhibitory (−)	Increased (↑) Decreased (↓)
PCC	dACC	−	↑
rHP	PCC	−	↑
dACC	mPFC	−	↑
dACC	lAI	−	↑
rAMG	rDLPFC	+	↓
rAI	mPFC	−	↑
lAI	rHP	−	↑
lDLPFC	mPFC	+	↓
lDLPFC	dACC	+	↓
mPFC	mPFC	−	↑
lAI	lAI	−	↓
lHP	lHP	−	↓
rHP	rHP	−	↑

The excitatory influence of the lDLPFC was reduced on mPFC and dACC post rTMS as compared to pre rTMS. mPFC, which is the main hub node of DMN, was influenced by lDLPFC, dACC and rAI. The connectivity from lDLPFC to mPFC was reduced while connectivity from dACC to mPFC and rAI to mPFC was increased. dACC is also affecting lAI through increased inhibition. The dACC was influenced by PCC in the form of increased inhibition. Also, the excitatory connection from rHP to PCC was decreased. Four of the nodes namely mPFC, lAI and bilateral HP also had self-connections. In DCM, self-connections are always inhibitory; after rTMS, the self-inhibition of these regions increased except lHP and lAI (as mentioned in Table 2), making them more resistant to the incoming influences from other regions. Overall, we found decreased strength of excitatory connections and increased strength of inhibitory connections among extrinsic connections. All parameters are reported in Supplementary Table S4.

### Connectivity difference between post-rTMS and pre-rTMS in sham group

The connectivity differences between pre- and post-rTMS were also found in sham group. There were a large number of connections which showed differences in post-rTMS in the sham group as compared to the active group (Supplementary Fig. S3). There were 8 common connections in active and sham groups showing differences in post-rTMS (5 between-regions and 3 self-connections) as shown in Supplementary Table S5. Among the common connections, the excitatory dACC to mPFC and rHP to PCC connections were reduced while in active group, they represent enhance inhibitory connections post-rTMS. The inhibitory connectivity from lDLPFC to mPFC and dACC increased while in active group, they were the reduced excitatory connections in post-rTMS condition. Interestingly, the strength of all the excitatory and inhibitory connections decreased and increased respectively as it did in the active group. These connectivity patterns perhaps indicate the placebo effect that the overall excitatory influence decreased while the overall inhibitory influence increased between the regions after sham treatment.

### Association of post-rTMS and pre-rTMS effective connectivity with emotional health

**Fig. 3:**
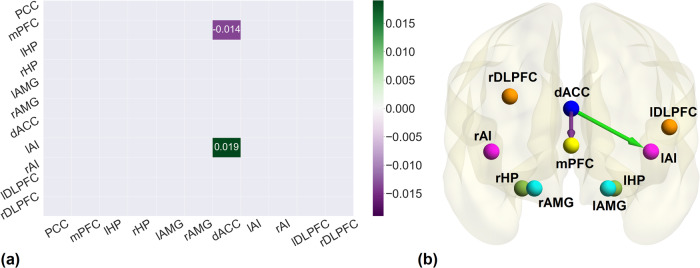
Association between post-rTMS effective connectivity and emotional health. **a** The association matrix between post-rTMS connectivity of active group and emotional health scores. The purple gradient indicates negative association while green gradient shows positive association. **b** The brain diagram of the association between post-rTMS effective connectivity of active group and emotional health. Green and Purple arrows illustrate the positive and negative association respectively. All the associations reported here survived the threshold of posterior probability >0.95 amounting to a strong evidence.

In the post-rTMS active group association analysis, using PEB, between effective connectivity and the emotional health scores, resulted in two connections surviving the threshold of *pp* > 0.95 (Fig. 3). We found dACC to mPFC to be negatively associated and dACC to lAI was positively associated with the behavioral scores. Same analysis performed for post-rTMS sham group yielded no association of emotional health scores with any connection.

The association analysis of pre-rTMS connectivity with emotional health scores was performed (Supplementary Fig. S4). The analysis revealed 21 connections before the treatment while there were only 2 connections after the treatment (active group only) that are associated with the emotional health scores. We found dACC to mPFC to be positively associated with behavioral scores at the pre-rTMS condition.

## Discussion

This is the first study that employed effective connectivity analysis after rTMS treatment in veterans with mild TBI. The analysis was performed using spectral DCM over a cortico-limbic network comprising 11 regions of interest that are most vulnerable to the injury. The regions include core hubs of anterior and posterior DMN i.e. mPFC and PCC respectively, medial temporal lobe (hippocampus), SN (bilateral AMG, dACC, bilateral AI), and executive network (bilateral DLPFC). These are the regions that are mostly discussed with respect to emotional processing. The purpose of our study was to discover underlying neural mechanisms of the emotional health improvement in veterans with TBI after providing rTMS therapy. The effective connectivity changes in active and sham group post-rTMS were analyzed. The connectivity changes found in the sham group were suspected due to the placebo effect which was further clarified by finding no associativity between the connections and emotional health data. Therefore, our focus is mainly on the active group effective connectivity changes and their possible interpretations.

It has been previously shown that the rTMS when delivered to lDLPFC, induces antidepressant effects in patients by altering the connectivity of cortico-limbic regions [59–61]. In the current study, it was hypothesized that the lDLPFC influences dACC by enhancing the inhibition after rTMS. Previous studies have shown that there was diminished functional connectivity between dACC and DLPFC during late-life depression which could not resolve using medication [62] while TMS was able to alter the activity of ACC when applied to DLPFC in healthy or depressed subjects [63–66]. The structural changes in ACC are also known to accompany TMS application on lDLPFC [67]. In a study with healthy participants, only the connectivity of the network containing dACC among 20 resting-state networks, was modulated by applying rTMS on lDLPFC [59] and the network comprised regions associated with depression. The dysfunction of prefrontal cortical regions account for the psychiatric disorders related to mood dysregulation [68, 69]. Moreover, it is known that DLPFC performs lateralized functioning during depression in the form of hyperactivity of right DLPFC and hypoactivity of left DLPFC [62, 70]. In our study, the inhibition of excitatory connection from rAMG to rDLPFC after rTMS highlights the importance of the inhibitory influence on rDLPFC which may affect emotional balance.

The amygdala is the subcortical limbic region known for its functions in emotion regulation and hence its connectivity has been widely studied during stress and depression which are common psychological disorders after TBI. Studies have shown that amygdala activity increases during emotional responses, including stress and anxiety disorder and rTMS is known to be effective in reducing the effect of these disorders. In chronic TBI, comorbid with depression, increased bilateral amygdala functional connectivity with several regions was reported [27]; also in acute TBI, increased amygdala connectivity with other brain regions was found [21]. We found decreased excitation from right amygdala to right DLPFC after rTMS which may reflect the overcoming of the increased effectivity connectivity in TBI patients as is also evident in the previous research [71] where trauma-exposed patients had increased effective connectivity from right amygdala to right DLPFC. Usually, it is expected that mPFC would control the amygdala activity via a top-down mechanism but in our case, there was no difference in the connectivity from mPFC to amygdala before and after applying rTMS which can be interpreted as no change in fear related emotion regulation involving amygdala after neuromodulation therapy.

In case of resting state, the role of salience network (SN) is broadly defined in interoception or self-awareness. dACC and AI are the parts of the SN which is known to guide behavior by integrating information from internal and external stimuli [72]. Anterior insula is a critical region for emotional awareness and self-reflection [73, 74] within SN. The right AI becomes activated during encoding of negative emotions [73, 75] which are energy consuming while left AI becomes activated during encoding of both negative and positive emotions [73]. The coactivation of AI and dACC is crucial for the processing of emotional functions [75, 76] and anterior insula is found to be functionally connected with anterior cingulate in the resting state as well [77]. Both regions form the input and output mechanism for the functional system producing subjective feelings [76]. The dACC performs a response initiation function for the integrated sensory inputs coming from AI. In major depressive disorder, the functional connectivity between AI and ACC is correlated with the severity level [78]. The activity of AI increased in anxious individuals [79] and the activity of ACC was increased in mTBI veterans [25]. According to our results, there is decreased excitation from dACC and PCC to lAI and dACC respectively which reflects the decreased excitatory influence on lAI and dACC post TBI after applying rTMS. Moreover, it suggests the presence of emotional awareness circuit in the subjects including the causal connection from rAI to mPFC. Furthermore, AI not only performs the sensory integration but also integration of bottom-up interoceptive information and top-down predictions during predictive coding of self-awareness [73], therefore, the increased connection from rAI to mPFC may indicate the improvement in the process of passing on the error signals in predictive coding of self-awareness.

In depression and anxiety, usually the symptoms are the result of negative or exaggerated self-referential processes. mPFC is a major hub of DMN which is usually activated during self-referential processes such as mentalizing and autobiographical thinking [80, 81]. DMN is engaged during internally oriented cognition such as the self-referential process, recalling the past, planning the future, and pondering upon others’ selves. It comprises interacting subsystems including anterior and posterior subsystems of mPFC and PCC respectively, and MTL subsystems [82]. This network is known to be activated during resting-state and deactivated during task performance [28, 83]. PCC and HP both play an active role in episodic memory processing and their interaction is critical for new memory formation and memory retrieval. Since, PCC is a major hub of DMN which is activated during internally-oriented tasks therefore, during memory encoding process that is encoding of external stimuli, the activity of DMN regions, including PCC, reduces while it increases during retrieval process which is internally oriented while the activity of hippocampus increases in both encoding and retrieval processes [84]. Though, hippocampus is usually considered to be part of DMN but during episodic encoding, it was found to have increased activity which differentiates it from other DMN regions and hence can be considered as a separate network during memory formation [84]. The two phases of episodic memory; encoding (i.e., forming of new memories) and recognition (i.e. retrieval of old memories) involves temporal lobes of left and right hemispheres respectively [85–87]. In MCI patients study, PCC activity was related with hippocampus activity during successful encoding and recognition of episodic memory [88]. In TBI subjects, the functional connectivity between HP and PCC was weaker than normal subjects [89, 90]. The connectivity between PCC and hippocampus is important for episodic memory processes. We found increased inhibitory effect of rHP over PCC post-rTMS compared to the pre-rTMS TBI patients. The directed connectivity from PCC to hippocampus was found to impair the episodic memory encoding [91], based on which we speculate that directionality for episodic memory function should be in the other direction as we have reported. Therefore, the directed connections in our analysis including the influence of rHP over PCC may also demonstrate the memory-related enhancement after rTMS which would eventually improve mental health. The posterior part of the DMN including PCC was found to have increased activity in TBI subjects [22]. In a previous study, the resting state functional connectivity of PCC with lDLPFC, dACC and bilateral insular cortex was found to be increased in chronic TBI [92]. Our study revealed increased inhibitory connectivity from PCC to dACC. Both regions, being the hubs of DMN and SN respectively, have significance in TBI. Coordination across networks, specifically DMN and SN, is essential not only for effective cognitive function [93] but also for efficient affective processing [94, 95]. It is previously shown that TBI results in aberrant interactions between the DMN and SN [96]. The increased functional connectivity between dACC and PCC during affective interference in depressive patients may signify limitations in switching between large-scale networks; when emotionally compelling but irrelevant to the task information is identified, depressed individuals may be unable to change their focus from internal to external world [94].

Overall, the influence of various brain regions to either increase inhibition or decrease excitation on dACC, AI, PCC and mPFC after rTMS suggests that there was an irregular self-referential behavior which includes over-thinking about the trauma that they had in the past. We suggest that the self-referential behavior was improved by using rTMS as a consequence of the compensation of the emotional circuit. Alternatively, it may be suggested that the removal of unwanted thoughts due to the trauma as an after-effect of neuromodulation therapy would result in mood improvement and emotional balance.

This hypothesis was further strengthened by a finding that the association between emotional health scores and the effective connectivity parameters post rTMS in active group provide evidence that the connections from dACC to mPFC and dACC to lAI were related to the improvement in emotional health. The connection from dACC to mPFC is negatively associated with the emotional health scores which signify that the diminished inhibitory effect of dACC to mPFC will yield better emotional health which is in accordance with the results of another study which reports that the cognitive control is inversely proportional to the dACC and mPFC functional connectivity [97]. In our analysis, it could be translated as controlling the urge by the participants to be influenced by negative emotions would enhance the emotional health. There was a positive association of dACC to lAI connection with emotional health scores and as discussed above, their connectivity is of vital importance for emotional regulation. The difference between the emotional health scores of active group pre- and post-rTMS were statistically significant with greater mean value for post-rTMS subjects showing emotional health improvement post rTMS, while that of sham subjects were not statistically significant. Moreover, there was no association of any effective connection in the sham group with emotional health data which elaborate the point made earlier that although there were connectivity changes in sham group after rTMS but they did not have any effect on emotional well-being. The association analysis of pre-rTMS connectivity with emotional health scores provided some interesting results. The analysis revealed 21 connections before the treatment while there were only 2 connections after the treatment (active group only) that are associated with the emotional health scores. Among the 21 connections, the connectivity from dACC to mPFC is common with the association analysis of the post-rTMS active group. The notable difference in this specific connection is that at the baseline the association was positive while after rTMS application, the same connectivity turned into a negative association with the emotional scores. The post-rTMS association analysis additionally highlighted the significance of the connectivity from dACC to lAI. Previous research has shown that rTMS modulates ACC connectivity with other brain regions when applied on lDLPFC resulting in reduced depression [98]. The association analysis also revealed that a number of connections important for emotional health were reduced after the therapeutic treatment of rTMS. We would like to point out that we only associated the effective connectivity with the emotional health scores at pre- and post-rTMS conditions. This is because the post- vs pre-rTMS changes in effective connectivity were estimated at the group level only hence precluding any analysis where we can associate the change in effective connectivity with the change in emotional health scores in post -vs pre-rTMS conditions. While this analysis of associating change in connectivity and behavioral scores would be useful, as it makes use of data from both pre and post conditions, however our simplified analysis of using only pre-rTMS and post r-TMS measures has the advantage of simpler interpretation.

## Conclusion

Our findings uncover the neural mechanisms underlying the improvement in emotional well-being in TBI due to application of neuromodulation. The main effect of rTMS is to reduce emotional disorders and hence consequently it may improve cognitive and executive functions. When rTMS was applied to lDLPFC, it helped in the emotional health improvement but failed to influence executive function (details on executive function in supplementary information). One of the reasons could be the target location of rTMS or the stimulation parameters. The limitation of this study includes the smaller dataset with only 32 subjects which were further divided into active and sham where all females were randomized into active treatment group. Therefore, there was no female participant in the sham group which may result in biased results. The inferred effective connectivity from this study needs to be further validated with a larger and balanced dataset.

### Supplementary information

Supplementary information

## Data Availability

The veterans’ MRI dataset is not publicly available but can be shared on a reasonable request to authors. The code for fMRI preprocessing, ICA-AROMA application, spectral dynamic causal modeling application, parametric empirical bayes for group level analysis and for associativity analysis is available at https://github.com/tjays7/TBI_TMS_emotional.

## References

[1] Goldstein M (1990). Traumatic brain injury: a silent epidemic. Ann Neurol.

[2] Assistant Secretary of Defense. Traumatic brain injury: updated definition and reporting. 2015. http://www.dcoe.mil/content/Navigation/Documents/DCoE.

[3] Masel BE, DeWitt DS (2010). Traumatic brain injury: a disease process, not an event. J Neurotrauma.

[4] Rao V, Lyketsos C (2000). Neuropsychiatric sequelae of traumatic brain injury. Psychosomatics..

[5] Emery CA, Barlow KM, Brooks BL, Max JE, Villavicencio-Requis A, Gnanakumar V (2016). A systematic review of psychiatric, psychological, and behavioural outcomes following mild traumatic brain injury in children and adolescents. Can J Psychiatry.

[6] Caeyenberghs K, Leemans A, Heitger MH, Leunissen I, Dhollander T, Sunaert S (2012). Graph analysis of functional brain networks for cognitive control of action in traumatic brain injury. Brain..

[7] Emilien G, Waltregny A (1996). Traumatic brain injury, cognitive and emotional dysfunction. Impact of clinical neuropsychology research. Acta Neurol Belg.

[8] Demirtas-Tatlidede A, Vahabzadeh-Hagh AM, Bernabeu M, Tormos JM, Pascual-Leone A (2012). Noninvasive brain stimulation in traumatic brain injury. J Head Trauma Rehabil.

[9] Marklund N, Bellander BM, Godbolt AK, Levin H, McCrory P, Thelin EP (2019). Treatments and rehabilitation in the acute and chronic state of traumatic brain injury. J Intern Med.

[10] Adamson M, Siddiqi S, Swaminath G, Wu L, McNerney W, Wortman K (2019). Repetitive transcranial magnetic stimulation for improving cognition in veterans with TBI: results from pilot clinical trial. Brain Stimul.

[11] Oberman LM, Exley S, Philip NS, Siddiqi SH, Adamson MM, Brody DL (2020). Use of repetitive transcranial magnetic stimulation in the treatment of neuropsychiatric and neurocognitive symptoms associated with concussion in military populations. J Head Trauma Rehabil.

[12] Herrold AA, Siddiqi SH, Livengood SL, Bender Pape TL, Higgins JP, Adamson MM (2020). Customizing TMS applications in traumatic brain injury using neuroimaging. J Head Trauma Rehabil.

[13] Mayer AR, Ling JM, Allen EA, Klimaj SD, Yeo RA, Hanlon FM (2015). Static and dynamic intrinsic connectivity following mild traumatic brain injury. J Neurotrauma.

[14] McDonald BC, Saykin AJ, McAllister TW (2012). Functional MRI of mild traumatic brain injury (mTBI): Progress and perspectives from the first decade of studies. Brain Imaging Behav.

[15] Medaglia JD (2017). Functional neuroimaging in traumatic brain injury: from nodes to networks. Front Neurol.

[16] Sharp DJ, Scott G, Leech R (2014). Network dysfunction after traumatic brain injury. Nat Rev Neurol.

[17] Bonnelle V, Leech R, Kinnunen KM, Ham TE, Beckmann CF, de Boissezon X (2011). Default mode network connectivity predicts sustained attention deficits after traumatic brain injury. J Neurosci.

[18] Wu SCJ, Jenkins LM, Apple AC, Petersen J, Xiao F, Wang L (2020). Longitudinal fMRI task reveals neural plasticity in default mode network with disrupted executive-default coupling and selective attention after traumatic brain injury. Brain Imaging Behav.

[19] van der Horn HJ, Scheenen ME, de Koning ME, Liemburg EJ, Spikman JM, van der Naalt J (2017). The default mode network as a biomarker of persistent complaints after mild traumatic brain injury: a longitudinal functional magnetic resonance imaging study. J Neurotrauma.

[20] Lancaster K, Venkatesan UM, Lengenfelder J, Genova HM (2019). Default mode network connectivity predicts emotion recognition and social integration after traumatic brain injury. Front Neurol.

[21] Iraji A, Benson RR, Welch RD, O’Neil BJ, Woodard JL, Ayaz SI (2015). Resting state functional connectivity in mild traumatic brain injury at the acute stage: independent component and seed-based analyses. J Neurotrauma.

[22] Zhou Y, Milham MP, Lui YW, Miles L, Reaume J, Sodickson DK (2012). Default-mode network disruption in mild traumatic brain injury. Radiology.

[23] Sharp DJ, Beckmann CF, Greenwood R, Kinnunen KM, Bonnelle V, De Boissezon X (2011). Default mode network functional and structural connectivity after traumatic brain injury. Brain..

[24] De Simoni S, Grover PJ, Jenkins PO, Honeyfield L, Quest RA, Ross E (2016). Disconnection between the default mode network and medial temporal lobes in post-traumatic amnesia. Brain.

[25] Sheth C, Rogowska J, Legarreta M, McGlade E, Yurgelun-Todd D (2021). Functional connectivity of the anterior cingulate cortex in Veterans with mild traumatic brain injury. Behav Brain Res.

[26] Moreno-López L, Sahakian BJ, Manktelow A, Menon DK, Stamatakis EA (2016). Depression following traumatic brain injury: a functional connectivity perspective. Brain Inj.

[27] Han K, Chapman SB, Krawczyk DC (2015). Altered amygdala connectivity in individuals with chronic traumatic brain injury and comorbid depressive symptoms. Front Neurol.

[28] Raichle ME (2015). The brain’s default mode network. Annu Rev Neurosci.

[29] Jorge RE, Robinson RG, Moser D, Tateno A, Crespo-Facorro B, Arndt S (2004). Major depression following traumatic brain injury. Arch Gen Psychiatry.

[30] Dailey NS, Smith R, Vanuk JR, Raikes AC, Killgore WDS (2018). Resting-state functional connectivity as a biomarker of aggression in mild traumatic brain injury. Neuroreport.

[31] Howlett JR, Nelson LD, Stein MB (2022). Mental health consequences of traumatic brain injury. Biol Psychiatry.

[32] Terri T, Jaycox LH, eds. Invisible Wounds of War: Psychological and Cognitive Injuries, Their Consequences, and Services to Assist Recovery. Santa Monica, CA: RAND Corporation; 2008.

[33] Hoy KE, Mcqueen S, Elliot D, Herring SE, Maller JJ, Fitzgerald PB (2019). A pilot investigation of repetitive transcranial magnetic stimulation for post-traumatic brain injury depression: safety, tolerability, and efficacy. J Neurotrauma.

[34] Lee SA, Kim MK (2018). Effect of low frequency repetitive transcranial magnetic stimulation on depression and cognition of patients with traumatic brain injury: a randomized controlled trial. Med Sci Monit.

[35] Rao V, Bechtold K, McCann U, Roy D, Peters M, Vaishnavi S (2019). Low-frequency right repetitive transcranial magnetic stimulation for the treatment of depression after traumatic brain injury: a randomized sham-controlled pilot study. J Neuropsychiatry Clin Neurosci.

[36] Siddiqi SH, Trapp NT, Hacker CD, Laumann TO, Kandala S, Hong X (2019). Repetitive transcranial magnetic stimulation with resting-state network targeting for treatment-resistant depression in traumatic brain injury: a randomized, controlled, double-blinded pilot study. J Neurotrauma.

[37] Siddiqi SH, Trapp NT, Shahim P, Hacker CD, Laumann TO, Kandala S (2019). Individualized connectome-targeted transcranial magnetic stimulation for neuropsychiatric sequelae of repetitive traumatic brain injury in a retired NFL player. J Neuropsychiatry Clin Neurosci.

[38] Stilling J, Paxman E, Mercier L, Gan LS, Wang M, Amoozegar F (2020). Treatment of persistent post-traumatic headache and post-concussion symptoms using repetitive transcranial magnetic stimulation: a pilot, double-blind, randomized controlled trial. J Neurotrauma.

[39] Siddiqi SH, Trapp NT, Shahim P, Hacker CD, Laumann TO, Kandala S (2019). Individualized connectome-targeted transcranial magnetic stimulation for neuropsychiatric sequelae of repetitive traumatic brain injury in a retired NFL player. J Neuropsychiatry Clin Neurosci.

[40] Friston KJ, Harrison L, Penny W (2003). Dynamical causal modelling. Neuroimage.

[41] Razi A, Friston KJ (2016). The Connected Brain: causality, models, and intrinsic dynamics. IEEE Signal Process Mag.

[42] Friston KJ, Kahan J, Biswal B, Razi AA (2014). DCM for resting state fMRI. Neuroimage..

[43] Razi A, Kahan J, Rees G, Friston KJ (2015). Construct validation of a DCM for resting state fMRI. Neuroimage..

[44] Almgren H, Van de Steen F, Kühn S, Razi A, Friston K, Marinazzo D (2018). Variability and reliability of effective connectivity within the core default mode network: a multi-site longitudinal spectral DCM study. Neuroimage..

[45] van der Horn HJ, Liemburg EJ, Aleman A, Spikman JM, van der Naalt J (2016). Brain networks subserving emotion regulation and adaptation after mild traumatic brain injury. J Neurotrauma.

[46] Bornhofen C, Mcdonald S (2008). Emotion perception deficits following traumatic brain injury: a review of the evidence and rationale for intervention. J Int Neuropsychol Soc.

[47] Hogeveen J, Salvi C, Grafman J (2016). ‘Emotional intelligence’: lessons from lesions. Trends Neurosci.

[48] Hays RD, Sherbourne CD, Mazel RM (1993). The RAND 36-item health survey 1.0. Health Econ.

[49] Ware JE. SF-36 health survey: manual and interpretation guide. Boston: Health Institute, New England Medical Center; 1993.

[50] Pruim RHR, Mennes M, van Rooij D, Llera A, Buitelaar JK, Beckmann CF (2015). ICA-AROMA: a robust ICA-based strategy for removing motion artifacts from fMRI data. Neuroimage.

[51] Calhoun VD, Adali T, Pearlson GD, Pekar JJ (2001). A method for making group inferences from functional MRI data using independent component analysis. Hum Brain Mapp.

[52] Shirer WR, Ryali S, Rykhlevskaia E, Menon V, Greicius MD (2012). Decoding subject-driven cognitive states with whole-brain connectivity patterns. Cereb Cortex.

[53] Tzourio-Mazoyer N, Landeau B, Papathanassiou D, Crivello F, Etard O, Delcroix N (2002). Automated anatomical labeling of activations in SPM using a macroscopic anatomical parcellation of the MNI MRI single-subject brain. Neuroimage..

[54] Zeidman P, Jafarian A, Corbin N, Seghier ML, Razi A, Price CJ (2019). A guide to group effective connectivity analysis, part 1: first level analysis with DCM for fMRI. Neuroimage..

[55] Razi A, Seghier ML, Zhou Y, McColgan P, Zeidman P, Park HJ (2017). Large-scale DCMs for resting-state fMRI. Netw Neurosci.

[56] Friston KJ, Litvak V, Oswal A, Razi A, Stephan KE, Van Wijk BCM (2016). Bayesian model reduction and empirical Bayes for group (DCM) studies. Neuroimage..

[57] Zeidman P, Jafarian A, Seghier ML, Litvak V, Cagnan H, Price CJ (2019). A guide to group effective connectivity analysis, part 2: second level analysis with PEB. Neuroimage..

[58] Xia M, Wang J, He Y (2013). BrainNet viewer: a network visualization tool for human brain connectomics. PLoS One.

[59] Tik M, Hoffmann A, Sladky R, Tomova L, Hummer A, Navarro de Lara L (2017). Towards understanding rTMS mechanism of action: stimulation of the DLPFC causes network-specific increase in functional connectivity. Neuroimage..

[60] Dichter GS, Gibbs D, Smoski MJ (2015). A systematic review of relations between resting-state functional-MRI and treatment response in major depressive disorder. J Affect Disord.

[61] Cash R, Zalesky A, Thomson RH, Tian Y, Cocchi L, Fitzgerald PB (2019). Subgenual functional connectivity predicts antidepressant treatment response to transcranial magnetic stimulation: independent validation and evaluation of personalization. Biol Psychiatry.

[62] Aizenstein HJ, Butters MA, Wu M, Mazurkewicz LM, Stenger VA, Gianaros PJ (2009). Altered functioning of the executive control circuit in late-life depression: episodic and persistent phenomena. Am J Geriatr Psychiatry.

[63] Baeken C, Marinazzo D, Wu GR, Van Schuerbeek P, De Mey J, Marchetti I (2014). Accelerated HF-rTMS in treatment-resistant unipolar depression: Insights from subgenual anterior cingulate functional connectivity. World J Biol Psychiatry.

[64] Paus T, Castro-Alamancos MA, Petrides M (2001). Cortico-cortical connectivity of the human mid-dorsolateral frontal cortex and its modulation by repetitive transcranial magnetic stimulation. Eur J Neurosci.

[65] Teneback CC, Nahas Z, Speer AM, Molloy M, Stallings LE, Spicer KM (1999). Changes in prefrontal cortex and paralimbic activity in depression following two weeks of daily left prefrontal TMS. J Neuropsychiatry Clin Neurosci.

[66] Tik M, Woletz M, Schuler AL, Vasileiadi M, Cash RFH, Zalesky A (2023). Acute TMS/fMRI response explains offline TMS network effects – an interleaved TMS-fMRI study. Neuroimage..

[67] Lan MJ, Chhetry BT, Liston C, Mann JJ, Dubin M (2016). Transcranial magnetic stimulation of left dorsolateral prefrontal cortex induces brain morphological changes in regions associated with a treatment resistant major depressive episode; an exploratory analysis. Brain Stimul.

[68] George MS, Ketter TA, Post RM (1994). Prefrontal cortex dysfunction in clinical depression. Depression.

[69] Meyer BM, Rabl U, Huemer J, Bartova L, Kalcher K, Provenzano J (2019). Prefrontal networks dynamically related to recovery from major depressive disorder: a longitudinal pharmacological fMRI study. Transl Psychiatry.

[70] Grimm S, Beck J, Schuepbach D, Hell D, Boesiger P, Bermpohl F (2008). Imbalance between left and right dorsolateral prefrontal cortex in major depression is linked to negative emotional judgment: an fMRI study in severe major depressive disorder. Biol Psychiatry.

[71] Chen F, Ke J, Qi R, Xu Q, Zhong Y, Liu T (2018). Increased inhibition of the amygdala by the mPFC may reflect a resilience factor in post-traumatic stress disorder: a resting-state fMRI granger causality analysis. Front Psychiatry.

[72] Menon V, Uddin LQ (2010). Saliency, switching, attention and control: a network model of insula function. Brain Struct Funct.

[73] Gu X, Hof PR, Friston KJ, Fan J (2013). Anterior insular cortex and emotional awareness. J Comp Neurol.

[74] Modinos G, Ormel J, Aleman A (2009). Activation of anterior insula during self-reflection. PLoS One.

[75] Craig AD (2011). Significance of the insula for the evolution of human awareness of feelings from the body. Ann NY Acad Sci.

[76] Medford N, Critchley HD (2010). Conjoint activity of anterior insular and anterior cingulate cortex: awareness and response. Brain Struct Funct.

[77] Taylor KS, Seminowicz DA, Davis KD (2009). Two systems of resting state connectivity between the insula and cingulate cortex. Hum Brain Mapp.

[78] Horn DI, Yu C, Steiner J, Buchmann J, Kaufmann J, Osoba A (2010). Glutamatergic and resting-state functional connectivity correlates of severity in major depression – the role of pregenual anterior cingulate cortex and anterior insula. Front Syst Neurosci.

[79] Alvarez RP, Kirlic N, Misaki M, Bodurka J, Rhudy JL, Paulus MP (2015). Increased anterior insula activity in anxious individuals is linked to diminished perceived control. Transl Psychiatry.

[80] Gusnard DA, Akbudak E, Shulman GL, Raichle ME (2001). Medial prefrontal cortex and self-referential mental activity: relation to a default mode of brain function. Proc Natl Acad Sci USA.

[81] Spreng RN, Grady CL (2010). Patterns of brain activity supporting autobiographical memory, prospection, and theory of mind, and their relationship to the default mode network. J Cogn Neurosci.

[82] Buckner RL, Andrews-Hanna JR, Schacter DL (2008). The Brain’s default network. Ann N Y Acad Sci.

[83] Raichle ME, MacLeod AM, Snyder AZ, Powers WJ, Gusnard DA, Shulman GL (2001). A default mode of brain function. Proc Natl Acad Sci USA.

[84] Huijbers W, Pennartz CMA, Cabeza R, Daselaar SM (2011). The hippocampus is coupled with the default network during memory retrieval but not during memory encoding. PLoS One.

[85] Jones-Gotman M, Zatorre RJ, Olivier A, Andermann F, Cendes F, Staunton H (1997). Learning and retention of words and designs following excision from medial or lateral temporal-lobe structures. Neuropsychologia..

[86] Kennepohl S, Sziklas V, Garver KE, Wagner DD, Jones-Gotman M (2007). Memory and the medial temporal lobe: hemispheric specialization reconsidered. Neuroimage.

[87] Tulving E, Kapur S, Craik FIM, Moscovitch M, Houle S (1994). Hemispheric encoding/retrieval asymmetry in episodic memory: positron emission tomography findings. Proc Natl Acad Sci USA.

[88] Papma JM, Smits M, de Groot M, Mattace Raso FU, van der Lugt A, Vrooman HA (2017). The effect of hippocampal function, volume and connectivity on posterior cingulate cortex functioning during episodic memory fMRI in mild cognitive impairment. Eur Radio.

[89] Irimia A, Maher AS, Chaudhari NN, Chowdhury NF, Jacobs EB (2020). Acute cognitive deficits after traumatic brain injury predict Alzheimer’s disease-like degradation of the human default mode network. GeroScience.

[90] Johnson B, Zhang K, Gay M, Horovitz S, Hallett M, Sebastianelli W (2012). Alteration of brain default network in subacute phase of injury in concussed individuals: resting-state fMRI study. Neuroimage.

[91] Natu VS, Lin JJ, Burks A, Arora A, Rugg MD, Lega B (2019). Stimulation of the posterior cingulate cortex impairs episodic memory encoding. J Neurosci.

[92] Sours C, Zhuo J, Roys S, Shanmuganathan K, Gullapalli RP (2015). Disruptions in resting state functional connectivity and cerebral blood flow in mild traumatic brain injury patients. PLoS One.

[93] Sridharan D, Levitin DJ, Menon V (2008). A critical role for the right fronto-insular cortex in switching between central-executive and default-mode networks. Proc Natl Acad Sci USA.

[94] Kaiser RH, Andrews-Hanna JR, Spielberg JM, Warren SL, Sutton BP, Miller GA (2015). Distracted and down: neural mechanisms of affective interference in subclinical depression. Soc Cogn Affect Neurosci.

[95] Hamilton JP, Chen MC, Gotlib IH (2013). Neural systems approaches to understanding major depressive disorder: an intrinsic functional organization perspective. Neurobiol Dis.

[96] Bonnelle V, Ham TE, Leech R, Kinnunen KM, Mehta MA, Greenwood RJ (2012). Salience network integrity predicts default mode network function after traumatic brain injury. Proc Natl Acad Sci USA.

[97] Fatfouta R, Meshi D, Merkl A, Heekeren HR (2018). Accepting unfairness by a significant other is associated with reduced connectivity between medial prefrontal and dorsal anterior cingulate cortex. Soc Neurosci.

[98] Schiena G, Franco G, Boscutti A, Delvecchio G, Maggioni E, Brambilla P. Connectivity changes in major depressive disorder after rTMS: a review of functional and structural connectivity data. Epidemiol Psychiatr Sci. 2021;30:e59.

