# Supplementary material for: Neural mechanisms of emotional health in traumatic brain injury patients undergoing rTMS treatment
Source: Mol Psychiatry. 2023 Jul 6;28(12):5150–8. doi: 10.1038/s41380-023-02159-z (PMC11041778; doi:10.1038/s41380-023-02159-z)
Supplement: Supplementary file 1 — Supplementary information [file 41380_2023_2159_MOESM1_ESM.docx]

### **Supplementary Information**

**Methods**

**General Protocol Specifications**

The experimental protocol was approved by the Institutional Review Boards of Veterans Affairs Palo Alto Health Care System (VAPAHCS) and Stanford University. All patients provided informed consent according to the Declaration of Helsinki. The study was registered

with ClinicalTrials.gov (NCT02152540). The study was monitored by mental health providers on an ongoing basis and rTMS stimulation was delivered by a trained nurse practitioner.

**Patients**

Thirty-three Veterans with TBI participated in the study. The majority of these were recruited from various departments in VAPAHCS such as War Related Illness and Injury Study Center (WRIISC), Polytrauma Systems of Care (PSC) and recruitment advertisements at surrounding VA clinics. Special permission was obtained from VA Rehabilitation Research office to recruit Veterans outside of California through various registries and VA wide advertisements. Airfare and hotel accommodations were made for these individuals and their caregivers.

Veterans first went through an initial telephone screen for inclusion and exclusion criteria including their neurological history such as the incidence of TBI, stroke, and seizure, chronic symptom status as well as rTMS and MRI safety concerns. After the Veteran signed the Informed Consent Form, the on-site screening procedures and assessments were initiated. Veterans were included based on: age 20-65 years; History of TBI (Post Traumatic Amnesia < 1 day for mild TBI; 1 day> x < 7 days for moderate TBI) based on VA/DOD criteria for TBI diagnosis; Ability to obtain a Resting Motor Threshold (MT) determined during the screening process; If on a psychotropic medication regimen, that regimen must have been stable for at least 4 weeks prior to entry to the study and patients were willing to remain on a table regimen during the acute treatment phase; Had an adequately stable condition and environment to enable attendance at scheduled clinic visits; and, were able to read, verbalize understanding, and voluntarily sign the Informed Consent Form prior to participating in any study-specific procedures or assessments. We excluded Veterans if they possessed any of the following characteristics: pregnant or lactating female; unable to be safely withdrawn, at least two-weeks prior to treatment commencement, from medications that substantially increase the risk of having seizures; had a cardiac pacemaker or a cochlear implant; had an implanted device (deep brain stimulation) or metal in the brain; had a mass lesion, cerebral infarct or other active CNS disease, including a seizure disorder; known current psychosis as determined by DSM-IV coding in chart (Axis I, psychotic disorder, schizophrenia) or a history of a non-mood psychotic disorder; diagnosis of Bipolar Affective Disorder (as determined by chart review and intake interview); current amnesic disorders, dementia, MMSE ≤ 24 or delirium; current substance abuse (not including caffeine or nicotine) as determined by positive toxicology screen, or by history via AUDIT, within 3 months prior to screening; prior history of seizures; severe TBI or open head injury; TBI within last two months or in acute stage; participation in another concurrent clinical trial; prior exposure to rTMS/ECT; and, active current suicidal intent or plan. Patients at risk for suicide were required to establish a written safety plan involving their primary psychiatrist and the treatment team before entering the clinical trial. All 33 Veterans met criteria for mild and moderate TBI based on the VA/DOD definition and a neurologist’s physical exam. Following screening and informed consent patients were randomized into one of two treatment groups: active or sham. Randomization to active or sham treatment was done by random number generator and mild and moderate TBI were used as variables. All patients went through baseline testing that collected demographic (including military history etc.), neuropsychological and self-report questionnaires for health problems.

Treatment Phase: After randomization, the rTMS administrator retested the resting motor threshold (MT). The exact location of motor cortex and DLPFC for rTMS target location was determined using the Visor2 LT 3D neuronavigation system (ANT Neuro, Netherlands) using T1-weighted anatomical MRI. Specifically, the resting fMRI activity of 38 healthy patients was used to identify the hubs of the default, executive control, dorsal attention, and salience networks in MNI space(1). The voxel of maximum activation for each respective hub was then used as the template target. For each subject, these template targets were warped to native space to render the target for accurate neuronavigated TMS based on left anterior middle frontal gyrus (aMFG) stimulation site within left DLPFC. The rTMS administrator then delivered DLPFC active rTMS treatment or sham (Control) rTMS treatment for 20 sessions. Active treatment was delivered using the MagventureTM X100 AC B65 cooling coil. Sham treatment was delivered using the same coil which produced a similar sound but does not deliver any stimulation. It is important to call it sham as the protocol was exactly the same except with no stimulation. Sham (Control) treatment: This system, or something quite similar, has been used in the rTMS and depression clinical trial (e.g. CSP556) and several other smaller studies in which the blinding has been maintained. Sham (Control) treatment was accomplished by using the Cool-B65-A/P coil that functions both as an active (A) and placebo (P) coil. It has a symmetrical mechanical design and no labeling on the coil indicates the active or placebo side. Consequently, it was not possible for the operator to see or hear which side is used. Additionally, for each treatment session, whether sham or active, each patient wore scalp electrodes through which, in the case of sham treatments, a low-voltage, low electric current (2 – 20mA at no more than 100V) passed in order to provide cutaneous stimulation that mimics the sensation of actual rTMS. At the same time, the Sham Noise Generator was used to hide the click noise produced by the rTMS. That is, when a magnetic stimulation pulse is fired, white noise was sent to the patient. This sham (white) noise hides the click noise from the participant (active or placebo). All other procedures, including neuronavigation, were identical for both groups. Treatment was administered over left DLPFC with intensity of 120% of motor threshold in 80 5-second trains at 10 Hz frequency with 10-second inter-train interval (20 sessions in total). The intensity of the rTMS stimulation was 120% of the motor threshold as determined by TMS motor threshold assessment tool. The entire acute treatment phase (20 sessions) normally took 2 weeks with 2-3 treatments each day. MRI scans were conducted at baseline and post-treatment. MRI scans and other self-report questionnaires were also completed within 2-5 hours of end of treatment.

Follow-up Phase: After the acute treatment phase ends, patients returned for a 6-month follow-up period. Patients were compensated for the entire rTMS treatment visit and additionally for the six month follow up MRI visit. (Note: Data from 6-month follow-up are not discussed here).

**Spectral Dynamic Causal Modelling**

Dynamic causal modelling (DCM) is Bayesian framework that infers the directed (causal) connectivity among the neuronal systems – referred to as effective connectivity. We recently proposed a new DCM for resting state fMRI – based upon a deterministic model that generates predicted cross spectra – referred to as spectral DCM. In order to model resting state activity – in the absence of external stimuli – we will have to add a stochastic component, i.e. neural fluctuations, to the classical DCM based on ordinary differential equations. Mathematically, we can express the formulation of the stochastic generative model using a set of two equations. First is the neuronal state equation, namely

$\dot{x}\left( t \right)=f\left( x(t),u(t),\theta\right)+ v(t)$, (S1)

and second is the observation equation, which is a static nonlinear mapping from the hidden physiological states in (S1) to the observed BOLD activity and is written as:

$y\left( t \right)=h\left( x(t),\varphi\right)+ e\left( t \right),$ (S2)

where $\dot{x}(t)$ is the rate of change of the neuronal states $x\left( t \right)$, $\theta$ are unknown parameters (i.e. the effective connectivity) and $v(t)$ (resp. $e(t)$) is the stochastic process – called the state noise (resp. the measurement or observation noise) – modelling the random neuronal fluctuations that drive the resting state activity. In the observation equations, $\varphi$ are the unknown parameters of the (haemodynamic) observation function and $u(t)$ represents any exogenous (or experimental) inputs that drive the hidden states – that are usually absent in resting state designs.(2) Spectral DCM furnishes a constrained inversion of the stochastic model by parameterising the neuronal fluctuations$v(t)$. Spectral DCM simplifies the generative model by replacing the original timeseries with their second-order statistics (i.e., cross spectra). This means, instead of estimating time varying hidden states, we are estimating their covariance which is time invariant. Then we simply need to estimate the covariance of the random fluctuations; where a scale free (power law) form for the state noise (resp. observation noise) is used – motivated from previous work on neuronal activity(3–5)– as follows:

$g_{v}\left( \omega,\theta\right)=\alpha_{v}\omega^{-\beta_{v}}$

$g_{e}\left( \omega,\theta\right)=\alpha_{e}\omega^{-\beta_{e}}$ (S3)

Here, $\left\{ \alpha,\beta\right\}\subset\theta$ are the parameters controlling the amplitudes and exponents of the spectral density of the neural fluctuations. The parameterisation of endogenous fluctuations means that the states are no longer probabilistic; hence the inversion scheme is significantly simpler, requiring estimation of only the parameters (and hyperparameters) of the model. We used standard Bayesian model inversion to infer the parameters of the model in (S1), (S2) and (S3), from the observed signal$y(t)$. The description of the Bayesian model inversion procedures based on variational Laplace can be found elsewhere for the interested readers.(6–8)

**Parametric Empirical Bayes**

Empirical Bayes refers to the Bayesian inversion or fitting of hierarchical models. In hierarchical models, constraints on the posterior density over model parameters at any given level are provided by the level above. These constraints are called empirical priors because they are informed by empirical data. We recently introduced a second-level or between-subjects model over parameters, which represents how individual (within-subject) connections derive from the subjects’ group membership(9) – based on parametric empirical Bayes (PEB). This approach calls on Bayesian Model Reduction (BMR) to finesse the inversion of multiple models of a single dataset or a single (hierarchical) model of multiple datasets. BMR allows one to compute posterior densities over model parameters, under new prior densities, without explicitly inverting the model again. For example, one can invert a DCM for each subject in a group and then evaluate the posterior density over group effects, using the posterior densities over parameters from the single subject inversion. This may improve subject-specific parameter estimates, by using group-level estimates to rescue individual DCM from local optima. Mathematically, for DCM studies with *N* subjects and *M* parameters per DCM, we have a hierarchical model, where the responses of the *i*-th subject and the distribution of the parameters over subjects can be modelled as:

$y_{i}=\Gamma_{i}^{\left( 1 \right)}(\theta^{\left( 1 \right)})+ \varepsilon_{i}^{\left( 1 \right)}$ (S4)

$\theta^{\left( 1 \right)}=\Gamma^{\left( 2 \right)}\left( \theta^{\left( 2 \right)} \right)+ \varepsilon^{\left( 2 \right)}$

$\theta^{\left( 2 \right)}=\eta+ \varepsilon^{\left( 3 \right)}$

where, $y_{i}$is the BOLD time series from *i-th* subject *and* $\Gamma_{i}^{\left( 1 \right)}$ is a nonlinear mapping from the parameters of a model to the predicted response $y$ for e.g. as shown in Eq. S1 above. $\varepsilon_{i}^{(1)}$is independent and identically distributed (i.i.d.) observation noise (equivalent to $e\left( t \right)$ in Eq. S2). In this hierarchical form, *empirical priors* encoding second (between-subject) level effects place constraints on subject-specific parameters. The second level would be a linear model where the random effects are parameterised in terms of their precision:

$\Gamma^{\left( 2 \right)}\left( \theta^{\left( 2 \right)} \right)=(X\bigotimes W)\beta$

where, $\beta\subset\theta$ are group means or effects encoded by a design matrix with between $X$ and within-subject $W$parts. The between-subject part encodes differences among subjects or covariates such as age, while the within-subject part specifies mixtures of parameters that show random effects. We assume that the first column of the design matrix is a constant term, modelling group means and subsequent columns encode group differences or covariates such as age.

**Effect of rTMS on executive functions**

The three brain networks we focused on; namely DMN, SN, and EN are mostly discussed together. DMN is considered a task-negative network and EN a task-positive network as DMN remains deactivated during task performance while EN is activated during task and vice versa for resting-state. SN provides the dynamical switching between these two networks during task and resting-state. The right anterior insula (rAI) is a causal outflow hub that regulates the switching between DMN and executive network (EN) as part of the salience network (SN) in both task-based and task-free paradigms in healthy subjects.(10) The structural and functional connectivity of rAI and dACC is critical for regulating the DMN activity and any abnormality in this connection due to TBI consequently leads to inefficient cognitive functions.(10,11) In a DCM study, the effective connectivity of working memory networks was explored during resting state. Their results demonstrated that dACC monitors the activity of DLPFC and hence influences executive functions.(12) Mild cognitive impairment (MCI) disrupts the interaction between DMN, EN, and dorsal SN; EN modulates the connections between DMN and SN instead of SN deriving the DMN and EN and it is suggested that this disruption is the consequence of neuronal changes which are associated with MCI.(13) We also tested the effect of rTMS therapy on the executive functions. The alternative hypothesis that the scores of Trail Making Test-B would improve after applying neuromodulation failed as the null hypothesis could not be rejected (p = 0.4233). We also applied PEB to find the association between the post-rTMS connectivity and Trail Making Test-B which is a neuropsychological test for execution functions. There found to be no association between them, hence, none of the differential connections had an effect on executive functions.

**References**

1. Chen AC, Oathes DJ, Chang C, Bradley T, Zhou ZW, Williamsa LM, et al. Causal interactions between fronto-parietal central executive and default-mode networks in humans. Proc Natl Acad Sci U S A [Internet]. 2013 Dec 3 [cited 2020 Nov 2];110(49):19944–9. Available from: /pmc/articles/PMC3856839/?report=abstract

2. Friston KJ, Kahan J, Biswal B, Razi A. A DCM for resting state fMRI. Neuroimage. 2014 Jul 1;94:396–407.

3. Beggs JM, Plenz D. Neuronal Avalanches in Neocortical Circuits. J Neurosci [Internet]. 2003 Dec 3 [cited 2022 Jul 28];23(35):11167–77. Available from: https://www.jneurosci.org/content/23/35/11167

4. Shin CW, Kim S. Self-organized criticality and scale-free properties in emergent functional neural networks. Phys Rev E Stat Nonlin Soft Matter Phys [Internet]. 2006 [cited 2022 Jul 28];74(4 Pt 2). Available from: https://pubmed.ncbi.nlm.nih.gov/17155118/

5. Stam CJ, De Bruin EA. Scale-free dynamics of global functional connectivity in the human brain. Hum Brain Mapp [Internet]. 2004 Jun [cited 2022 Jul 28];22(2):97–109. Available from: https://pubmed.ncbi.nlm.nih.gov/15108297/

6. Razi A, Friston KJ. The Connected Brain: Causality, models, and intrinsic dynamics. IEEE Signal Process Mag [Internet]. 2016 May 1 [cited 2020 Apr 17];33(3):14–55. Available from: http://ieeexplore.ieee.org/document/7461023/

7. Friston KJ, Harrison L, Penny W. Dynamic causal modelling. Neuroimage. 2003 Aug 1;19(4):1273–302.

8. Friston K, Mattout J, Trujillo-Barreto N, Ashburner J, Penny W. Variational free energy and the Laplace approximation. 2006 [cited 2020 Sep 25]; Available from: www.elsevier.com/locate/ynimg

9. Friston KJ, Litvak V, Oswal A, Razi A, Stephan KE, Van Wijk BCM, et al. Bayesian model reduction and empirical Bayes for group (DCM) studies. Neuroimage. 2016 Mar 1;128:413–31.

10. Sridharan D, Levitin DJ, Menon V. A critical role for the right fronto-insular cortex in switching between central-executive and default-mode networks. Proc Natl Acad Sci U S A [Internet]. 2008 Aug 26 [cited 2020 Nov 2];105(34):12569–74. Available from: www.pnas.org/cgi/content/full/

11. Bonnelle V, Ham TE, Leech R, Kinnunen KM, Mehta MA, Greenwood RJ, et al. Salience network integrity predicts default mode network function after traumatic brain injury. Proc Natl Acad Sci U S A [Internet]. 2012 Mar 20 [cited 2020 Oct 18];109(12):4690–5. Available from: https://pubmed.ncbi.nlm.nih.gov/22393019/

12. Fang X, Zhang Y, Zhou Y, Cheng L, Li J, Wang Y, et al. Resting-State Coupling between Core Regions within the Central-Executive and Salience Networks Contributes to Working Memory Performance. Front Behav Neurosci. 2016 Feb 25;0(FEB):27.

13. Chand GB, Wu J, Hajjar I, Qiu D. Interactions of the Salience Network and Its Subsystems with the Default-Mode and the Central-Executive Networks in Normal Aging and Mild Cognitive Impairment. Brain Connect [Internet]. 2017 Sep 1 [cited 2020 Nov 2];7(7):401–12. Available from: https://pubmed.ncbi.nlm.nih.gov/28707959/


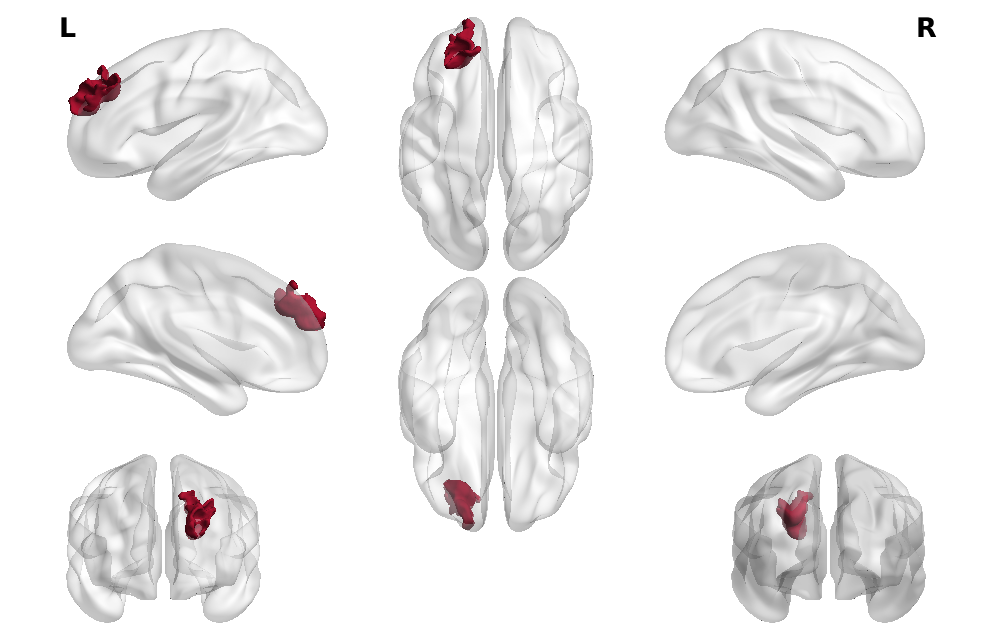
**Figure S1** **Depiction of the stimulation target (lDLPFC)**


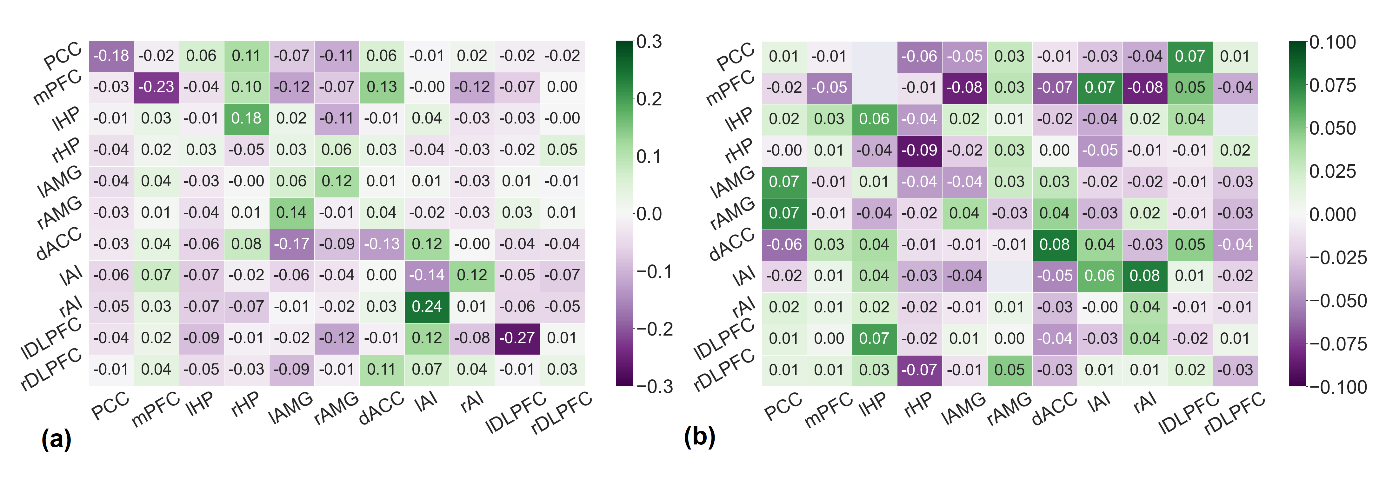
**Figure S2** **Mean connectivity matrices of active and sham groups (a)** The connectivity matrix illustrates mean connectivity of sham group over pre- and post-rTMS. **(b)** The connectivity matrix illustrates mean connectivity of active group over pre- and post-rTMS.

In both figures (a) and (b), the rows and columns represent the brain regions. The positive and negative values in the legend denote the valence of information flow i.e. excitation and inhibition respectively.


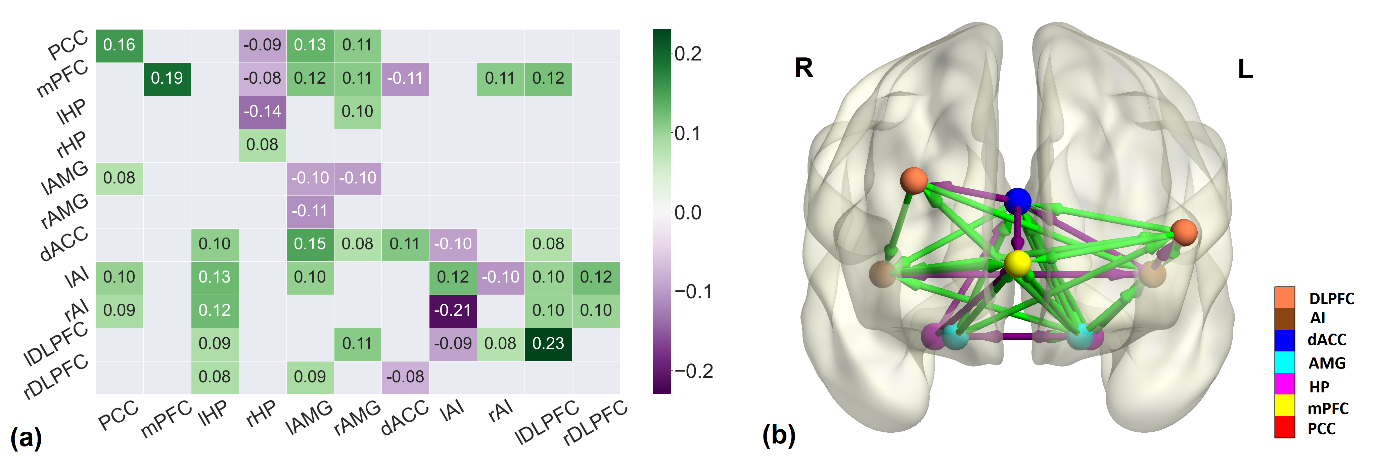


**Figure S3** **Effective connectivity changes in sham group after treatment with rTMS** **(a)** The connectivity matrix depicting difference in connectivity in Post-rTMS vs. Pre-rTMS Sham group. Rows and columns represent the brain regions. The positive and negative values in the legend denote the increase and decrease in connectivity Post-rTMS as compared to Pre-rTMS. **(b)** The same matrix depicted on a brain template. Green arrows show increased connectivity while purple arrows show decreased connectivity

**
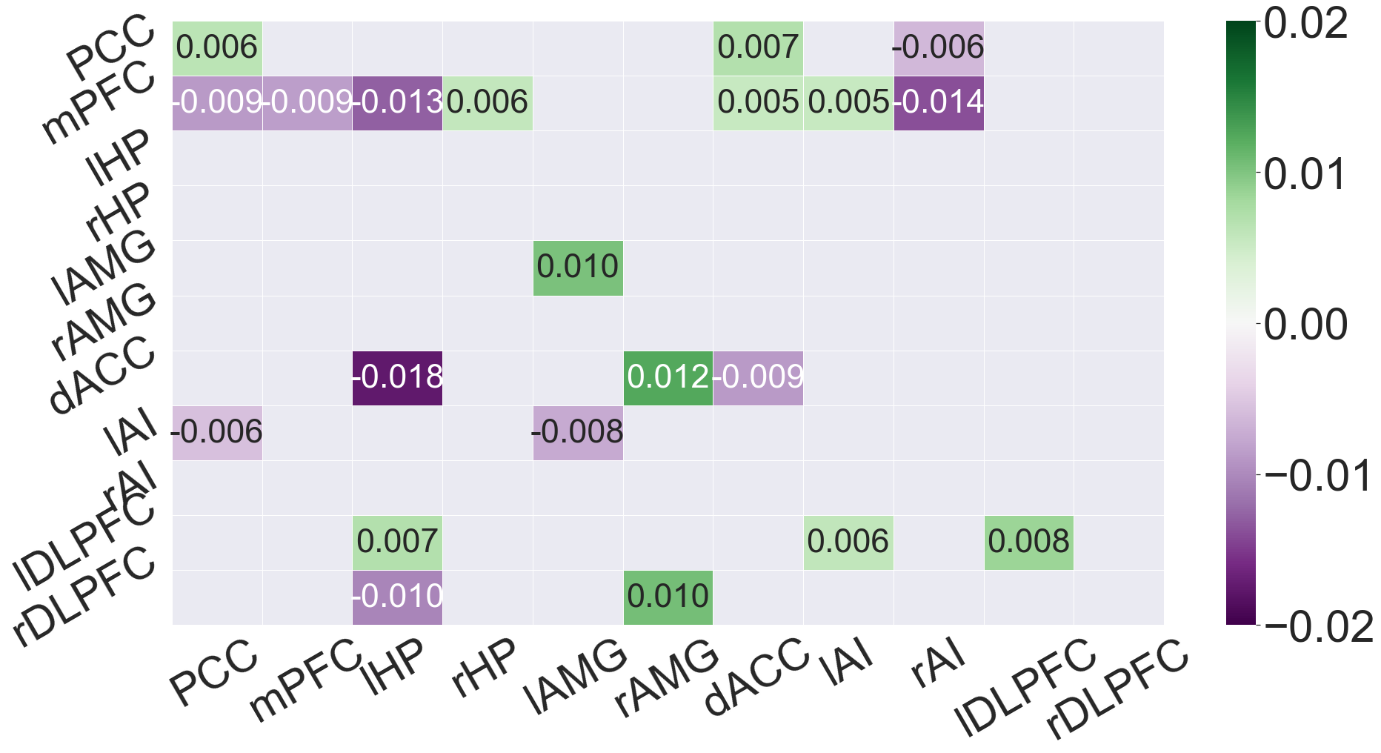
**

**Figure S4. Association between pre-rTMS effective connectivity and emotional health** The association matrix between pre-rTMS connectivity of the combined active and sham groups and emotional health scores. The purple gradient indicates negative association while green gradient shows positive association. All the associations reported here survived the threshold of posterior probability > 0.95 amounting to a strong evidence.

**Table S1. Items of the emotional health scale of VR-36**

| VR-36 item number | Description |
| --- | --- |
| 8b | Nervous |
| 8c | Nothing could cheer you up |
| 8d | Calm and peaceful |
| 8f | Downhearted |
| 8h | Happy |

**Table S2. Responses and their codes for emotional health scale of VR-36**

| Response | Code |
| --- | --- |
| All of the time | 1 |
| Most of the time | 2 |
| A good bit of the time | 3 |
| Some of the time | 4 |
| A little of the time | 5 |
| None of the time | 6 |

**Table S3. Coded and re-coded response values for emotional health scale of VR-36**

| VR-36 item number | Original response | Coded values |
| --- | --- | --- |
| 8b, 8c, 8f | 1  2  3  4  5  6 | 0  20  40  60  80  100 |
| 8f, 8h | 1  2  3  4  5  6 | 100  80  60  40  20  0 |

**Table S4. Parameters related to Connectivity Differences Between Pre and Post rTMS in Active Group**

| From | To | Mean | Variance | Posterior Probability |
| --- | --- | --- | --- | --- |
| PCC | dACC | 0.0994 | 0 | 1.0 |
| rHC | PCC | 0.0935 | 0 | 1.0 |
| dACC | mPFC | 0.1042 | 0 | 1.0 |
| dACC | lAI | 0.0897 | 0 | 1.0 |
| rAMG | rDLPFC | -0.0765 | 0 | 1.0 |
| rAI | mPFC | 0.0685 | 0 | 1.0 |
| lAI | rHC | 0.0792 | 0 | 1.0 |
| lDLPFC | mPFC | -0.0867 | 0 | 1.0 |
| lDLPFC | dACC | -0.0783 | 0 | 1.0 |
| mPFC | mPFC | 0.092 | 0 | 1.0 |
| lAI | lAI | -0.0889 | 0 | 1.0 |
| lHC | lHC | -0.1009 | 0 | 1.0 |
| rHC | rHC | 0.0741 | 0 | 1.0 |

|  | Active Post rTMS – Active Pre rTMS | | Sham Post rTMS – Sham Pre rTMS | |
| --- | --- | --- | --- | --- |
| Connection | **Excitatory (+)**  **Inhibitory (-)** | **Increased (🡩)**  **Decreased (🡫)** | **Excitatory (+)**  **Inhibitory (-)** | **Increased (🡩)**  **Decreased (🡫)** |
| dACC -> mPFC | - | 🡩 | + | 🡫 |
| rHP -> PCC | - | 🡩 | + | 🡫 |
| rAI -> mPFC | - | 🡩 | - | 🡩 |
| lDLPFC -> mPFC | + | 🡫 | - | 🡩 |
| lDLPFC -> dACC | + | 🡫 | - | 🡩 |
| lAI -> lAI | - | 🡫 | - | 🡩 |
| mPFC -> mPFC | - | 🡩 | - | 🡩 |
| rHP -> rHP | - | 🡩 | - | 🡩 |

**Table S5. Common Connectivity Differences in Active and Sham Groups**
